# Multidrug-Resistant Tuberculosis in Patients for Whom First-Line Treatment Failed, Mongolia, 2010–2011

**DOI:** 10.3201/eid2108.141860

**Published:** 2015-08

**Authors:** Claudia C. Dobler, Sarah Korver, Ochirbat Batbayar, Batiargal Nyamdulam, Sodnomdarjaa Oyuntsetseg, Bold Tsolmon, Bazarragchaa Surmaajav, Byambaa Bayarjargal, Ben J. Marais

**Affiliations:** Author affiliations: University of New South Wales, Sydney, New South Wales, Australia (C.C. Dobler); NHMRC Center of Research Excellence in Tuberculosis Control, University of Sydney, Sydney (C.C. Dobler, B.J. Marais); National Centre for Communicable Diseases, Ulaanbaatar, Mongolia (S. Korver, O. Batbayar, B. Nyamdulam, S. Oyuntsetseg, B. Tsolmon, B. Surmaajav, B. Bayarjargal); Marie Bashir Institute for Infectious Diseases and Biosecurity, Sydney (B.J. Marais)

**Keywords:** tuberculosis, multidrug-resistant tuberculosis, treatment failure, category I treatment, drug susceptibility testing, new sputum smear-positive patients, antimicrobial resistance, bacteria, respiratory infections, tuberculosis and other mycobacteria, Mongolia

## Abstract

In Ulaanbaatar, Mongolia, multidrug-resistant tuberculosis (MDR TB) was diagnosed for more than a third of new sputum smear–positive tuberculosis patients for whom treatment had failed. This finding suggests a significant risk for community-acquired MDR TB and a need to make rapid molecular drug susceptibility testing available to more people.

In many resource-limited settings, the high cost and technical complexity of drug susceptibility testing (DST) preclude its routine use for patients in whom sputum smear–positive tuberculosis (TB) has been newly diagnosed. This lack of testing is particularly problematic in settings in which prevalence of multidrug-resistant (MDR) TB (resistant to at least isoniazid and rifampin) is high. Delayed diagnosis and inappropriate treatment prolong the patient’s interval of infectiousness and decrease the prospect of treatment success (*1*)*.* Treating MDR TB with inappropriate drug regimens also increases the risk of amplifying drug resistance (*2**–**5*).

In Mongolia, failure of standard first-line TB treatment among patients with diagnosed MDR TB increased from 12% in 2006 to 38% in 2012 (Mongolian National TB Program [NTP], unpub. data; *6*). During the same period, the proportion of new TB patients with MDR TB increased from 0% to 17%. Although these findings partly reflect the implementation of improved MDR TB case-finding strategies, they may also reflect increased MDR TB transmission within the community. In this study, we aimed to determine the prevalence of MDR TB among new sputum smear–positive patients for whom first-line treatment failed and to evaluate factors associated with an increased risk for MDR TB among these patients. 

## The Study

Mongolia’s Guidelines on Tuberculosis Care and Services recommend DST for patients with newly diagnosed TB when they remain sputum smear-positive after 3 months of TB treatment, when they are in close contact with someone with drug-resistant TB, or when they are co-infected with HIV (Ministry of Health Mongolia, World Health Organization, Global Fund Supported Project on AIDS and TB, unpub. data). We performed a retrospective cohort study of all new sputum smear–positive patients who began directly observed therapy for TB in Ulaanbaatar during 2010 or 2011. HIV-infected patients and those with close contact with MDR TB patients were excluded.

About 45% of the population of Mongolia lives in Ulaanbaatar, the country’s capital. TB cases were reported to Mongolia’s NTP database from 9 districts of Ulaanbaatar, a prison hospital, and a hospital for the homeless. Cases that were subsequently diagnosed as MDR TB were identified from the NTP MDR TB database. Cases were excluded from the analysis if standard first-line treatment was altered for any reason.

New sputum smear–positive TB cases were defined as cases in patients who had >1 acid-fast bacillus in >1 sputum sample and who had never received TB treatment before. These patients were given routine first-line treatment, consisting of isoniazid, rifampin, pyrazinamide, and ethambutol during a 2-month intensive phase followed by isoniazid and rifampin during a 4-month continuation phase. MDR TB was diagnosed if the *Mycobacterium tuberculosis* strain isolated was resistant to at least isoniazid and rifampin. Treatment outcomes were defined according to World Health Organization definitions (*6*).

Sputum samples were processed at the Mongolia National Reference TB Laboratory, which used solid cultures (Löwenstein-Jensen medium and Ogawa) and BACTEC Mycobacteria Growth Indicator Tube 960 (Becton, Dickinson and Company, Franklin Lakes, NJ, USA) liquid cultures during the study period. Phenotypic DST was performed by using the BACTEC Mycobacteria Growth Indicator Tube and Löwenstein-Jensen medium (*7*,*8*). Susceptibility to isoniazid, rifampin, ethambutol, and streptomycin was tested. The Xpert MTB/RIF assay (Cepheid, Sunnyvale, CA, USA) was not available in Mongolia before 2013.

In total, 1,920 new sputum smear–positive patients were identified during the study period; 45 were excluded from the analysis because they did not receive standard first-line treatment because of adverse drug effects or drug shortages. Table 1 summarizes the demographic characteristics of all 1,875 patients included in the study. Among these patients, 476 (25%) were employed; 63 (3%) were homeless, and 50 (3%) were prisoners. The median age was 31 years (range 12–97 years).

**Table 1:** Characteristics of patients with new sputum smear–positive TB and those for whom first-line treatment failed

Characteristics	Patients with new TB cases, no. (%)	Patients for whom TB treatment failed, no. (%)*	Patients with MDR TB, no. (%)†
All	1,875 (100)	156 (8.3)	54 (34.6)
Sex			
M	1,071 (57.1)	96 (9.0)	27 (28.1)
F	804 (42.9)	60 (7.5)	27 (45.0)
Age, y			
<15	10 (0.5)	2 (20.0)	1 (50.0)
15–34	1,097 (58.5)	82 (7.5)	38 (46.3)
35–54	611 (32.6)	61 (10.0)	14 (23.0)
≥55	155 (8.3)	11 (7.1)	1 (9.1)
Missing	2 (0.1)	0	0
Occupation			
Employed, including self-employed	476 (25.4)	29 (6.1)	16 (55.2)
Unemployed	774 (41.3)	68 (8.8)	19 (27.9)
Retired	123 (6.6)	11 (8.9)	1 (9.1)
Student‡	240 (12.8)	15 (6.3)	11 (73.3)
School-age§	58 (3.1)	6 (10.3)	1 (16.7)
On disability pension	70 (3.7)	8 (11.4)	1 (12.5)
In prison	50 (2.7)	8 (16.0)	1 (12.5)
Homeless	63 (3.4)	10 (15.9)	3 (30.0)
Unknown	21 (1.1)	1 (4.8)	1 (100)
Treatment facility/district			
Bayangol	222 (11.8)	15 (6.8)	10 (66.7)
Bayanzurkh	392 (20.9)	52 (13.3)	14 (26.9)
Songinokhairkhan	426 (22.7)	28 (6.6)	7 (25.0)
Sukhbaatar	203 (10.8)	10 (4.9)	4 (40.0)
Khan-Uul	176 (9.4)	17 (9.7)	9 (52.9)
Chingeltei	261 (13.9)	13 (5.0)	6 (46.2)
Prison hospital	50 (2.7)	8 (16.0)	1 (12.5)
Enerel, hospital for the homeless	63 (3.4)	10 (15.9)	3 (30.0)
Other¶	82 (4.4)	3 (3.7)	0

*Percentage of patients with new sputum smear–positive TB. †MDR TB, multidrug–resistant tuberculosis (resistant to isoniazid and rifampin). Percentage of patients for whom treatment failed. ‡Student, enrolled in higher education or vocational training. §School-age, enrolled in primary or secondary school. ¶Districts with <50 new sputum smear–positive patients (Baganuur, Nalaikh, Bagakhangai).

Successful treatment outcomes fell short of World Health Organization targets. A total of 1,436 (77%) patients were cured, and 102 (5%) completed treatment (*6*), but for 156 (8%) patients, first-line treatment failed. An additional 34 (2%) patients were transferred out (i.e., transferred to a different reporting unit with unknown treatment outcome), 41 (2%) died, and 106 (6%) interrupted treatment for >2 consecutive months. Treatment failure rates were highest among adults 35–54 years of age, prisoners, and those who were homeless (Table 1).

Among the 1,875 new sputum smear–positive case-patients, MDR TB was diagnosed for 66 (4%). Of these 66 patients, treatment failure was designated for 54 (82%). Therefore, of the 156 total patients for whom first-line treatment failed, 54 (35%) had MDR TB. Bivariate analysis showed that MDR TB among patients in whom first-line treatment failed was significantly associated with being female (odds ratio [OR] 2.1, 95% CI 1.1–4.1), <35 years of age (OR 3.3, 95% CI 1.6–6.7), and employed or a student (OR 5.1, 95% 2.4–10.8) (Table 2). These associations remained significant after adjusting for sex, age, and occupation (Table 2). For 32 (59%) MDR TB patients for whom first-line treatment failed, complete resistance to all 4 first-line drugs tested (isoniazid, rifampin, ethambutol, and streptomycin) was found (Technical Appendix Table).

**Table 2:** Bivariate and multivariate analysis of factors associated with multidrug-resistant TB among patients for whom first-line tuberculosis treatment failed

Characteristic	MDR TB cases/treatment failures*	Unadjusted odds ratio (95% CI)	p value	Adjusted odds ratio (95% CI)†	p value
Sex					
M	27/96	1.00 (Reference)		1.00 (Reference)	
F	27/60	2.09 (1.06-4.11)	0.032	2.19 (1.01-4.74)	0.047
Age, y					
≥35	15/72	1.00 (Reference)		1.00 (Reference)	
<35	39/84	3.29 (1.62-6.71)	0.001	2.42 (1.11-5.27)	0.026
Occupation					
Unemployed, prisoner, homeless	25/105	1.00 (Reference)		1.00 (Reference)	
Employed, student	27/44	5.08 (2.39-10.81)	<0.001	4.59 (2.04-10.31)	<0.001

*MDR TB, multidrug-resistant tuberculosis. †Multivariate analysis adjusted for gender, age, and occupation. Persons <18 years of age were excluded when we adjusted for occupation.

## Conclusions

In Ulaanbaatar, MDR TB was diagnosed for more than a third of sputum smear–positive patients in whom standard first-line TB treatment failed. Resistance against all first-line drugs tested was found for ≈60% of these patients. This finding suggests successful transmission of these highly resistant strains, as has been documented in other MDR TB–endemic areas (*9*). A high level of streptomycin resistance among patients in whom first-line treatment has failed indicates that use of the standard retreatment regimen prolongs the duration of ineffective treatment and should be abandoned. Apart from poor patient outcomes and the risk for ongoing TB transmission, continued use of an inadequate treatment regimen encourages the amplification of drug resistance.

Ideally, universal DST would be offered at the time of diagnosis, but in the absence of sufficient resources, increased use of rapid molecular diagnostics should be considered. Despite implementation hurdles, the Xpert MTB/RIF assay rapidly confirms *M. tuberculosis* infection and assesses resistance to rifampin without the need for extensive laboratory infrastructure (*10*). Rapid testing should be considered for all patients in whom MDR TB is suspected, including those for whom sputum smears did not convert to negative after 2–3 months of first-line treatment. Prompt initiation of appropriate therapy should improve patient outcomes and reduce ongoing MDR TB transmission within the community. In this context, the addition of spatial information and accurate mapping of MDR TB hot spots within Mongolia may guide targeted strategies for early detection and treatment for MDR TB.

Our study does have limitations. DST was performed at the discretion of the treating physician, and detection bias may have influenced MDR TB risk factor analyses. Because not everyone was tested, the reported MDR TB rate represents a minimum estimate. Without DST results from specimens collected before treatment initiation, we cannot provide definite proof of transmitted (primary) MDR TB. However, although a patient can acquire MDR TB after 2–3 months of TB treatment, acquisition of MDR TB is unlikely if quality-assured multidrug treatment and directly observed therapy are used. The conclusion that most cases represented primary MDR TB not detected when the patient originally sought treatment is further supported by the high rate of resistance against all first-line drugs. Comparison with previous drug resistance surveys indicates that the proportion of MDR TB cases among new sputum smear–positive patients increased from 1.0% (4/405) during 1998–99 (*11*) to 1.4% (9/650) in 2007 (*12*) to 3.4% (66/1920) in our study. This increase in transmitted drug-resistant TB requires closer scrutiny and concerted global action (*13*).

Technical Appendix Phenotypic resistance to first-line drugs among multidrug-resistant tuberculosis patients for whom first-line treatment failed.
