# Supplementary material for: Multidrug-Resistant Tuberculosis in Patients for Whom First-Line Treatment Failed, Mongolia, 2010–2011
Source: Emerg Infect Dis. 2015 Aug;21(8):1451–4. doi: 10.3201/eid2108.141860 (PMC4517706; doi:10.3201/eid2108.141860)
Supplement: Technical Appendix — Phenotypic resistance to first-line drugs among multidrug-resistant tuberculosis patients for whom first-line treatment failed. [file 14-1860-Techapp-s1.pdf]

Article DOI: http://dx.doi.org/10.3201/eid2108.141860

Multidrug-resistant Tuberculosis in Patients in Whom First-line Treatment Failed, Mongolia, 2010–2011

Technical Appendix

Methods

Study Setting and Study Population

Despite rapid economic growth, 27% of Mongolia's population lived in poverty in 2012, a key factor sustaining the TB epidemic (*1*). While the TB incidence in Mongolia decreased from 152/100,000 in 2003 to 141/100,000 in 2012, the incidence of diagnosed multidrug-resistant TB (MDR-TB) has risen from 1.5/100,000 in 2003 to 7.5/100,000 in 2012 (based on data from the Mongolian National TB Program). The prevalence of human immunodeficiency virus (HIV) infection, and TB/HIV co-infection, is low in Mongolia (*2*) with an estimated HIV prevalence of less than 0.1% among the adult population (*3*) and a reported TB/HIV co-infection incidence of 0.21 (0.2 to 0.51) per 100,000 in 2013 (*4*).

DOTS (directly observed therapy, short course) was established in Mongolia following a pilot project supported by the WHO Global Tuberculosis Programme in 1995. The implementation of DOTS is supported by the provision of community-DOT by a network of over 300 community volunteers coordinated by the Mongolian Anti-Tuberculosis Association (MATA) in addition to conventional clinic-based DOT.

Definitions

Cured was defined as a pulmonary TB patient with bacteriologically confirmed TB at the beginning of treatment who was smear- or culture-negative in the last month of treatment and on at least one previous occasion. Treatment completed was defined as a TB patient who completed treatment without evidence of failure, but with no record to show that sputum smear or culture results in the last month of treatment and on at least one previous occasion were negative, either because tests were not done or because results are unavailable.

Treatment failed was defined as a TB patient whose sputum smear or culture is positive at month five or later during treatment. Died was defined as a TB patient who dies for any reason before starting or during the course of treatment. Lost to follow-up was defined as a TB patient who did not start treatment or whose treatment was interrupted for two consecutive months or more. Not evaluated was defined as a TB patient for whom no treatment outcome is assigned. This includes cases “transferred out” to another treatment unit as well as cases for which the treatment outcome is unknown to the reporting unit. Treatment success was defined as the sum of cured and treatment completed. “Occupation” was defined based on categories included in the Mongolian routine TB data surveillance, which were employed (including self-employed), unemployed, retired, schoolchild (primary and secondary education), student (higher education and vocational training), people on disability pension, prisoner, homeless.

Cultures and Drug Susceptibility Testing

DST procedures passed an external quality assessment by the Japanese Research Institute of Tuberculosis (RIT), which acts as the supranational reference laboratory (SRL) for the Mongolian National Reference Laboratory (NRL).

DST to pyrazinamide was not performed during the study period (despite the use of pyrazinamide as part of the standard Category I treatment).

Analysis

Associations between demographic and clinical characteristics were assessed using logistic regression and odds ratios (ORs) with 95% confidence intervals (95% CIs). The independent effect of potential predictors of MDR-TB among patients in whom Category I treatment failed, was estimated using multivariate logistic regression. Only categorical variables were used in logistic regression; the continuous variable age was converted into a categorical variable by creating age groups. Multivariate analysis included adjustment for age, sex and occupation. Districts/treatment facilities were excluded from multivariate analysis to prevent overcorrection, as they had markedly different populations in terms of gender and age distribution as well as level of unemployment. Additionally, the study was underpowered to detect a significant association between the nine different districts/facilities and the risk of MDR-TB. Statistical significance was defined as $p < 0.05$. The dichotomous

outcome variable included all TB cases subsequently diagnosed with MDR-TB compared with all TB cases not diagnosed with MDR-TB, potentially including cases with mono- and poly-resistant TB. Statistical analysis was performed using SPSS Statistics 22 (IBM, Armonk, NY, USA) and Microsoft Excel 2010 (Microsoft Corporation, Redmond, WA, USA).

Ethical Considerations

As data were collected as part of routine TB surveillance, this analysis was not considered research involving human subjects, and ethics approval was not required.

Results

Of 66/1920 (3%) MDR-TB cases diagnosed among new sputum smear-positive cases, 54/66 (82%) were diagnosed among treatment failures and 12 (18%) among patients who either died (n=2), were lost to follow-up (n=1), transferred out (n=1) or were "cured" according to the WHO definition but later had recurrent disease (either relapse or reinfection) (n=8).

Two 14-year-old children with sputum smear-positive disease were treatment failures. MDR-TB was subsequently confirmed in one while in the other treatment failed despite completing a full 6-months course of standard Category I treatment; no DST was performed.

Table. Phenotypic resistance to first-line drugs among MDR-TB cases identified from patients in which first-line treatment failed

MDR resistance pattern	No. (%)
HR	16 (29.6)
HRE	3 (5.6)
HRS	3 (5.6)
HRES	32 (59.3)
Total	54 (100)

*MDR-TB= multidrug-resistant tuberculosis; H= isoniazid; R= rifampicin; E= ethambutol; S= streptomycin

References

1. The World Bank. Mongolia—World Development Indicators. [Cited 2014 Nov 1.] http://data.worldbank.org/country/mongolia

2. Davaalkham J, Unenchimeg P, Baigalmaa C, Oyunbileg B, Tsuchiya K, Hachiya A, et al. High-risk status of HIV-1 infection in the very low epidemic country, Mongolia, 2007. Int J STD AIDS. 2009;20:391–4. PubMed http://dx.doi.org/10.1258/ijsa.2008.008376

3. World Health Organization Western Pacific Region. Mongolia—HIV/AIDS [cited 2014 Nov 2.] http://www.wpro.who.int/mongolia/topics/hiv_aids/en/

4. World Health Organization. Tuberculosis country profiles—Mongolia [cited 2014 Nov 6.] http://www.who.int/tb/country/data/profiles/en/
